# Laser vs. bipolar prostate vaporization in bleeding-prone patients: a randomized trial and cutting-edge analysis

**DOI:** 10.1007/s00345-025-05692-4

**Published:** 2025-05-23

**Authors:** Ahmed A. Shorbagy, Mohammed Ismail, Youssef M. Kotb, Mohamed Desouki, Mohamed Shabayek, Peter Hanna

**Affiliations:** 1 Department of Urology, Ain Shams University, Cairo, Egypt; 2 Department of Urology, Aswan University, Aswan, Egypt

**Keywords:** Bipolar electro vaporization, Diode laser, Bleeding tendency, Anticoagulant

## Abstract

**Purpose:**

To compare effectiveness and safety of transurethral diode laser vaporization of prostate (diode LVP) versus bipolar transurethral vaporization of prostate (B-TUVP) in symptomatic benign prostatic hyperplasia (BPH) patients receiving oral anticoagulants/anti-platelet drugs.

**Patients and methods:**

All symptomatic prostate patients receiving oral anticoagulants/anti-platelet drugs are prospectively enrolled in the study between January 2023 to May 2024 in our institution. Of total 98 patients were randomized to B-TUVP (48 patients) or diode LVP (50 patients). The primary outcome is to compare B-TUVP with diode LVP regarding operative bleeding and postoperative hemoglobin drop. Secondary outcomes assess time of urinary irrigation, time of urethral catheter removal, length of hospital stays, and 6-month postoperative functional outcomes; IPSS, postoperative flow rate, and postvoid residual urine.

**Results:**

Baseline characteristics were similar in both groups. Diode LVP group had a significantly higher postoperative hemoglobin with a lower drop compared to B-TUVP group (p = 0.032, p = 0.007; respectively). The diode LVP group had a significantly reduced urinary irrigation duration compared to the B-TUVP group (p = 0.031). Also, diode LVP patients had early catheter removal compared to those in the B-TUVP group (p = 0.014). Additionally, the diode group had a notably reduced hospital stay duration compared to the B-TUVP group (p = 0.024). There were no significant differences between both groups regarding 6-month postoperative IPSS, Q max and PVRU.

**Conclusion:**

Diode LVP of the prostate is a safer alternative for treating BPH with fewer risks compared to B-TUVP especially in patients receiving anticoagulants. Nonetheless, both treatments yield similar 6-months functional outcomes.

**Supplementary Information:**

The online version contains supplementary material available at 10.1007/s00345-025-05692-4.

## Introduction

Benign prostatic hyperplasia (BPH) prevalence increases with age, affecting a significant portion of older males requiring treatment [1]. Transurethral resection of the prostate (TURP) remains the standard surgical approach; however, concerns exist regarding perioperative bleeding [2], particularly in patients with risk factors like diabetes, anticoagulation, and constipation [3].

The potential for complications such as prolonged catheterization, rehospitalization for bleeding, and electrolyte imbalances has increased interest in modern surgical technologies. Consequently, bipolar and laser energy modalities are being increasingly adopted for prostate tissue resection, vaporization, ablation, or enucleation [4–6].

In recent years, laser technologies have taken over the field of prostate resection, particularly for high-risk patients prone to bleeding or those on anticoagulants. The primary laser methods in use today include LBO (lithium borate), Diode lasers, Holmium yttrium–aluminum-garnet laser (Ho-YAG), Thulium fiber laser (TFL), and Thulium YAG (Tm-YAG) [7].

They offer remarkable hemostatic capabilities owing to their significant absorption by hemoglobin, yet their low water absorption results in slow ablative features, leading to extended operation duration [8]. The semiconductor diode laser is considered superior for its hemostatic characteristics; however, its acceptance was diminished due to postoperative dysuria, discomfort, and urinary storage symptoms [9]. A newly launched diode laser, functioning at a wavelength of 980 nm, showcases distinct characteristics through its innovative fiber design, enhanced tissue ablative properties, and effective hemostasis resulting from its notable concurrent absorption in water and hemoglobin [10].

The Biolitec LEONARDO"dual 200 device emits wavelengths of 980 nm and 1470 nm. It is designed to combine excellent coagulation with tissue ablation, making it suitable for treating benign prostatic hyperplasia with transurethral vaporization of the prostate [9].

Therefore, we aimed, in our study, to compare two different modalities in a prospective randomized fashion: Transurethral prostate vaporization with diode laser (diode LVP) versus bipolar transurethral vaporization of prostate (B-TUVP) in patients taking anticoagulant/anti-platelet medications.

## Patients and methods

### Study population

This prospective, randomized controlled trial, conducted at Ain Shams University Hospitals (Egypt) from January 2023 to May 2024, evaluated the short-term efficacy and safety of diode LVP versus B-TUVP in patients on anti-coagulant/anti-platelet medications with BPH (prostate size < 60 gm) requiring prostatectomy, over a 6-month postoperative follow-up. Inclusion and exclusion criteria are listed in the supplementary Table 1. All prostatic patients who met the inclusion criteria in the timeline from January 2023 to May 2024 were enrolled in this study.

Additionally, all our patients underwent standard laboratory tests and PSA screening, as well as pelvic ultrasound, to evaluate prostate size or perform a prostatic biopsy if necessary. Patients on antiplatelets (100 mg aspirin) continued therapy perioperatively without bridging. Oral anticoagulants (warfarin) were stopped 5 days pre-surgery and bridged with LMWH (40 mg/day) starting 2 days after, paused 12 h pre- and post-surgery, then overlapped with resumed warfarin until target INR. The study underwent rigorous ethical review and oversight, commencing with initial approval from the local Institutional Review Board (FWA 000017585). Continuous monitoring of each study phase was conducted by the committee, culminating in final approval upon verification of strict adherence to the approved protocol.

### Randomization

Participants were equitably distributed between the diode laser vaporization of the prostate (diode LVP) and bipolar transurethral vaporization of the prostate (B-TUVP) treatment arms via a 1:1 randomization protocol, facilitated by an online randomization tool (www.randomizer.at).

### Operative procedure

Operative procedures of bipolar electrovaporization were described previously [11]. The other group of patients were subjected to the 980-nm 200-W high-power diode laser vaporization. We used a 200-W machine (LEONARDO Dual 200, Biolitec, Germany) to generate a 980-nm laser through a diode semiconductor.

### Outcomes

The primary outcome is to compare two techniques (B-TUVP vs. diode LVP) in the treatment of BPH regarding post-operative bleeding in term of postoperative hemoglobin drop.

Secondary outcomes assess the time of urinary irrigation (hours), time of urethral catheter removal (days), length of hospital stays (days), and 6-month postoperative functional outcomes (IPSS, postoperative flow rate, and PVRU).

### Statistical analysis

All comparisons were performed between categorical variables using the Chi-square test and Fisher’s exact test and between continuous variables using the student’s *t* test if parametric and Mann–Whitney test if non-parametric. The comparison between two paired groups regarding quantitative data and parametric distribution was done by using a *Paired t test****.*** SPSS v.25 (IBM Corp, Armonk, NY, USA) was used to perform all statistical analyses. *P* values < 0.05 were taken to indicate statistical significance.

Sample size estimation was carried out utilizing PS Power and Sample Size Calculations Software (version 3.0.11 for MS Windows, William D. Dupont and Walton D. Vanderbilt, Department of Biostatistics, Vanderbilt University, Nashville, TN, USA). Prior to the initiation of this research, a randomized controlled trial (RCT) directly comparing (diode LVP) and (B-TUVP) in patients undergoing anticoagulant therapy was absent from the published literature. A clinically significant difference of 1-g in hemoglobin loss, designated as the primary outcome measure, was hypothesized [12]. A sample size of 40 participants per treatment arm was determined via Student's t test, employing a power of 90%, an alpha level of 0.05, and a 1:1 allocation ratio. To mitigate potential attrition and ensure adequate statistical power, the sample size was augmented by 20%, resulting in a final target of about 48 participants per group.

## Results

**Fig. 1:**
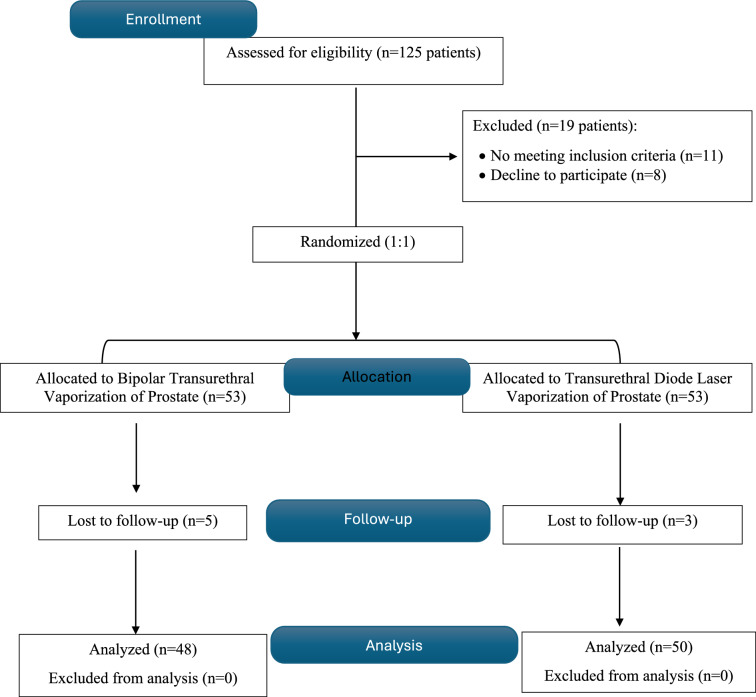
Flowchart for study participants

A prospective randomized study involved 98 patients with BPH. A total of 48 patients in the B-TUVP group and 50 patients in the diode LVP group were analyzed and compared. The CONSORT flow chart is shown in Fig. 1.

The baseline characteristics of both groups are listed in Table 1. There were no significant changes between both groups regarding age, preoperative IPSS, Q-Max, and PVRU.

**Table 1:** Comparison between bipolar and diode groups regarding demographic data and baseline characteristics of the patients studied

		Bipolar	Diode		P value
		No. = 48	No. = 50		
Age	Mean ± SD	60.96 ± 6.76	60.77 ± 6.24	0.916^a^	
	Range	50–72	50–69		
Prostate size (ml)	Mean ± SD	54.4 ± 4.2	51.7 ± 7.9	0.323^a^	
	Range	45–60	42–58		
Preoperative hemoglobin	Mean ± SD	12.46 ± 1.89	12.34 ± 3.75	0.968^a^	
	Range	9.3–15.6	9.5–15		
Charleson Comorbidity index				0.201^b^	
Mild (G1, 2)	No. (%)	14 (29.2%)	17 (34%)		
Moderate (G3, 4)	No. (%)	18 (37.5%)	20 (40%)		
Severe (≥ 5)	No. (%)	16 (33.3%)	13 (26%)		
Anticoagulant/Antiplatelet				0.921^b^	
LMWH	No. (%)	23 (47.9%)	24 (48%)		
Aspirin	No. (%)	25 (53.1%)	26 (52%)		
IPSS	Mean ± SD	23.76 ± 3.71	23.69 ± 3.42	0.932^a^	
	Range	17–32	16–29		
Q max	Mean ± SD	10.32 ± 1.42	9.75 ± 1.51	0.727^a^	
	Range	6.90–13.10	7.2–13.0		
PVRU	Mean ± SD	126.24 ± 45.77	118.44 ± 58.45	0.634^a^	
	Range	33–200	30–300		

P value > 0.05: Non-significant (NS); P value < 0.05: Significant (S); P value < 0.01: Highly significant (HS)

^*^Superscript letter (a) refers to comparisons using independent T test

^*^Superscript letter (b) refers to comparisons using Chi square test

### Postoperative outcomes

There was no significant difference between studied groups regarding preoperative hemoglobin level (P = 0.968), but post-hemoglobin was significantly higher in the diode group than B-TUVP group (P = 0.032) with hemoglobin drop being less in the diode group than in the B-TUVP group (P = 0.007). The diode LVP group showed a significantly shorter urinary irrigation time (mean ± SD 13.44 ± 11.32 h) compared to the B-TUVP group (mean ± SD 24.15 ± 16.43 h) (p = 0.031). Similarly, diode LVP patients showed early catheter removal (mean ± SD 1.34 ± 0.83 days) compared to the B-TUVP group (mean ± SD 2.46 ± 1.12 days) (p = 0.014). Also, the diode LVP group showed a significantly shortened length of hospital duration compared to the B-TUVP group (mean ± SD 2.31 ± 0.99 days vs. 3.65 ± 1.12 days; respectively) (p = 0.024) (Table 2).

**Table 2:** Postoperative outcomes in bipolar and diode patient groups

Postoperative outcomes		Bipolar	Diode	95% (CI) of mean difference		P value
		No. = 48	No. = 50	Lower	Upper	
Postoperative hemoglobin	Mean ± SD	10.34 ± 1.29	11.18 ± 1.47	– 1.33	0.29	0.032
	Range	9.20–14.40	9.50–14.20			
Hemoglobin drop	Mean ± SD	1.83 ± 1.23	0.81 ± 0.82	– 1.21	0.14	0.007
Urinary irrigation (hours)	Mean ± SD	24.15 ± 16.43	13.44 ± 11.32	– 3.86	11.25	0.031
	Range	12–48	12–48			
Urethral catheter removal/day	Mean ± SD	2.46 ± 1.12	1.34 ± 0.83	– 0.15	1.07	0.014
	Range	1–4	1–4			
Length of hospital stays (days)	Mean ± SD	3.65 ± 1.12	2.31 ± 0.99	– 0.15	1.08	0.024
	Range	2–5	2–5			
IPSS	Mean ± SD	8.23 ± 2.43	8.21 ± 3.29	– 1.72	1.49	0.886
	Range	5–14	1–16			
Q max	Mean ± SD	17.23 ± 1.75	18.45 ± 3.27	– 1.49	1.44	0.975
	Range	13–20	11–23			
PVRU	Mean ± SD	126.24 ± 45.77	37.39 ± 15.38	– 8.61	8.30	0.971
	Range	20–60	0–70			

P value > 0.05: Non-significant (NS); P value < 0.05: Significant (S); P value < 0.01: Highly significant (HS)

^*^All comparisons using independent T test

There were no significant differences between both groups regarding postoperative complications (supplementary Table 2).

Regarding functional outcomes, The IPSS and PVRU were significantly lower in 6 months postoperative compared to preoperative in B-TUVP and diode cases (p < 0.001, p < 0.001 respectively). Q max was significantly higher in 6 months postoperative compared to the baseline preoperative values in B-TUVP and diode case (p < 0.001; p < 0.001; respectively) (Table 3).

**Table 3:** preoperative and 6 months postoperative values of IPSS, Q-max and PVRU in bipolar patients vs. diode patients

		Bipolar (No. 48)		Differences	P value	Diode (No. 50)		Differences	P value
		Preoperative	6 months postoperative	Mean ± SD		Preoperative	6 months postoperative	Mean ± SD	
IPSS	Mean ± SD	23.76 ± 3.71	8.23 ± 2.43	– 15.53 ± 3.42	< 0.001	21.34 ± 3.75	8.21 ± 3.29	– 13.13 ± 3.98	< 0.001
	Range	17–32	5–14			16–29	1–16		
Q max	Mean ± SD	10.32 ± 1.42	17.23 ± 1.75	6.91 ± 1.55	< 0.001	9.75 ± 1.51	18.45 ± 3.27	8.70 ± 3.12	< 0.001
	Range	6.90–13.10	13–20			7.2–13	11–23		
PVRU	Mean ± SD	126.24 ± 45.77	33.47 ± 11.53	– 92.77 ± 46.24	< 0.001	118.44 ± 58.45	37.39 ± 15.38	– 81.05 ± 53.89	< 0.001
	Range	33–200	20–60			30–300	0–70		

P value > 0.05: Non-significant (NS); P value < 0.05: Significant (S); P value < 0.01: Highly significant (HS)

^*^All comparisons using independent T test

There were no significant differences between both groups regarding 6-month postoperative IPSS, Q max, and PVRU (p = 0.874, p = 0.921, p = 0.971; respectively) (supplementary Table 3).

## Discussion

Navigating the therapeutic landscape for symptomatic benign prostatic hyperplasia (BPH) presents a complex decision-making process for clinicians. The modalities now encompass medical therapy, minimally invasive procedures, TURP, laser surgery, and open surgery for the prostate. Key factors to consider in making treatment decisions are efficacy, long-lasting results, frequency of complications, length of hospital stay and catheter use, and cost analysis [13].

Technological advancements enable precise and effective prostate enucleation via various laser modalities offering resection, vaporization, or enucleation. Laser enucleation facilitates the complete resection of prostatic adenomas, irrespective of their dimensions with simultaneous hemostasis. However, widespread laser adoption, especially in developing countries, faces hurdles including cost, a significant learning curve, limited equipment, and insufficient expertise [14].

Authorized in 2007, the 980 nm diode laser has become popular for BPH treatment due to its potent vaporization and coagulation. Its simultaneous absorption by water and hemoglobin ensures efficient tissue ablation and effective hemostasis [15].

To our knowledge, it’s the first study comparing the efficacy of transurethral bipolar vaporization of prostate (B-TUVP) versus diode LVP for symptomatic BPH patients taking anti-coagulant/anti-platelet medications.

We prospectively compared two techniques in the treatment of BPH (diode-LVP using the Biolitec Leonardo dual 200 device diode laser and B-TUVP) concerning postoperative bleeding, safety, and efficacy (hospital stay, time of urethral catheter removal, flow rate, post-voiding residual urine, and IPSS) in patients with bleeding tendencies.

The diode LVP group of patients showed less bleeding, and less hemoglobin drop compared to the B-TUVP group. In the same context, Kai-Yi Tzou et al. showed significantly less hemoglobin drop in patients managed by diode laser with bipolar TURP compared to conventional monopolar TURP [16].

On the other hand, a randomized clinical trial conducted by Mohammad et al. showed no differences regarding postoperative hemoglobin level between the diode laser group and the TURP group [17].

That difference is attributed to that diode lasers exhibit simultaneous absorption in both water and hemoglobin, making them suitable for effective tissue ablation while ensuring superior hemostasis. Therefore, less bleeding is achieved especially in patients with anticoagulant therapy compared to B-TUVP as reported in our study. In a similar context, Chien-Hsu Che et al. concluded that diode laser prostatectomy can achieve excellent hemostasis, especially for those who were on anticoagulant therapy [18].

Boeri et al. retrospectively analyzed 438 patients on anticoagulant or antiplatelet medications who underwent prostate enucleation using either holmium laser (HoLEP, n = 296, 67.6%) or bipolar transurethral (B-TUEP, n = 142, 32.4%) techniques. They found that patients with and without anticoagulant/antiplatelet (AC/AP) therapy exhibited comparable outcomes regarding operative time, hemoglobin drop, 2-month IPSS, and transfusion rates [19].

In our study, patients managed by diode LVP showed shorter urinary irrigation time and early catheter removal compared to the B-TUVP group. Consequently, the diode LVP group showed early hospital discharge compared to the B-TUVP group.

Within this framework, a randomized clinical trial was conducted on 115 patients (50 patients managed by diode laser vs 52 patients managed by TURP). It showed a significantly less postoperative catheterization time and shortened length of hospital stays [17].

Sanjo et al. also showed comparable results between both groups (antithrombotic therapy vs. control group) in patients undergoing diode laser vaporization of prostate in terms of same median catheterization period (2 days) and the median hospital stay period (5 days) in both groups [20].

Regarding functional outcomes in terms of postoperative Q-max, PVRU, and 6-month IPSS, we didn’t report any significant differences between both groups.

Multiple studies reported similar functional outcomes between TURP and diode laser [17, 21].

Our study's primary strength lies in its novelty as the first prospective randomized controlled trial directly comparing diode LVP and B-TUVP specifically in bleeding-prone BPH patients on antithrombotic medications, a critical and understudied population. The findings that demonstrating significantly less postoperative bleeding and faster early recovery with diode LVP offer valuable initial evidence for its potential advantages in this high-risk group. This prospective comparison provides a crucial foundation for future research aimed at optimizing surgical management in this challenging clinical context.

Our study is not devoid of limitations. A modest sample size, lack of blinding or allocation concealment, the use of a per-protocol (PP) analysis as our primary approach, and shorter duration of follow-up are the major limitations of the study. Moreover, the lack of reporting preoperative coagulation profile was another shortcoming. Also, our study's findings are potentially limited by the absence of subgroup analysis for different anticoagulant and platelet inhibitor types, which could affect bleeding outcomes. Therefore, more randomized controlled trials with longer follow-up times and larger sample sizes are necessary.

## Conclusion

Our study revealed that diode LVP of the prostate is a safer and less invasive treatment option for BPH compared to B-TUVP, reducing postoperative complications such as hemoglobin drop, hematuria, irrigation time, catheterization time, and hospital stay. However, both procedures show comparable functional outcomes and appear to be equally effective for IPSS, Q-Max, and PVRU in treating BPH. Briefly, the use of a high-powered diode laser (980 nm) can be considered a safe and practical alternative for B-TUVP in treating symptomatic BPH, especially in patients receiving anticoagulant/antiplatelet medications.

## Supplementary Information

Below is the link to the electronic supplementary material.

Supplementary file1 (DOCX 20 KB)

## Data Availability

No datasets were generated or analysed during the current study.
