# Supplementary material for: Laser vs. bipolar prostate vaporization in bleeding-prone patients: a randomized trial and cutting-edge analysis
Source: World J Urol. 2025 May 23;43(1):322. doi: 10.1007/s00345-025-05692-4 (PMC12102097; doi:10.1007/s00345-025-05692-4)
Supplement: Supplementary file 1 — Supplementary file1 (DOCX 20 KB) [file 345_2025_5692_MOESM1_ESM.docx]

**Supplementary Material**

**Table (1):** Inclusion and exclusion criteria of patients

| **Inclusion criteria** | **Exclusion criteria** |
| --- | --- |
| - Patients with indication of prostatectomy (prostate size < 60 gm): - Patients with LUTS (voiding ± storage) refractory to medical TTT with IPSS >20 (International prostate scoring system) and Q max < 10 ml/s. - Refractory acute urinary retention. - Hematuria or recurrent urinary tract infections 2ry to BPH. - This patient also has one of these:   • Bleeding disorders  • Any risk for bleeding tendency.  • On anticoagulation drugs  • On antiplatelet drugs | ***Any patients with:***   - Prostate size more than 60 grams - Neurogenic bladder - Urinary bladder stone - Prostatic carcinoma - Urinary bladder carcinoma - Urethral stricture. - With previous bladder, urethral or prostate surgery - Unfit for anesthesia |

**Table (2):** Postoperative complications in both groups

| **Complications** | **Bipolar**  **(n=48)** | **Diode**  **(n=50)** | **P-value** |
| --- | --- | --- | --- |
| No significant complication | 32 (66.7 %) | 33 (66 %) | 0.7^*^ |
| Dysuria | 6 (12.5 %) | 7 (14 %) |  |
| Urgency | 1 (2.1 %) | 2 (4 %) |  |
| Frequency | 2 (4.2 %) | 0 (0.0 %) |  |
| Weak stream | 1 (2.1 %) | 3 (6 %) |  |
| Postoperative hematuria (moderate and severe ) | 6 (12.5 %) | 5 (10 %) |  |

* Fisher’s exact test

**Table (3):** Comparison between bipolar and diode groups regarding IPSS, Q-max and PVRU after 6 months of the studied patients

|  | | **Bipolar** | **Diode** | **P-value** | **Sig.** |
| --- | --- | --- | --- | --- | --- |
|  |  | **No. = 48** | **No. = 50** |  |  |
| IPSS after 6 months | Mean ± SD | 8.23 ± 2.43 | 8.21 ± 3.29 | 0.874^*^ | NS |
|  | Range | 5 – 14 | 1 – 16 |  |  |
| Q max after 6 months | Mean ± SD | 17.23 ± 1.75 | 18.45 ± 3.27 | 0.921^*^ | NS |
|  | Range | 13 – 20 | 11 – 23 |  |  |
| PVRU after 6 months | Mean ± SD | 33.47 ± 11.53 | 37.39 ± 15.38 | 0.971^*^ | NS |
|  | Range | 20 – 60 | 0 – 70 |  |  |

P-value > 0.05: Non-significant (NS) ; P-value < 0.05: Significant (S); P-value < 0.01: Highly significant (HS)

* All comparisons using independent T-test
